# Causal Effects Between Anxiety-Depressive and Subjective Tinnitus in Europe: A Bidirectional Mendelian Randomization Study

**DOI:** 10.1007/s12070-025-05618-x

**Published:** 2025-06-09

**Authors:** Cheng Zhong, Li-hua Wang, Ying Dong, Haopeng Zhang, Lin Ji, Yu Guo

**Affiliations:** 1 Department of Otolaryngology, Shanghai Municipal Hospital of Traditional Chinese Medicine, Shanghai University of Traditional Chinese Medicine, No.274, Zhijiang Middle Road, Jing’an District, Shanghai, China; 2 School of Public Health, Shanghai University of Traditional Chinese Medicine, Shanghai, China

**Keywords:** Tinnitus, Anxiety-depression, Mendelian randomization, Causality

## Abstract

This study aimed to explore the potential causal relationship between anxiety-depression and tinnitus using a bidirectional mendelian randomization (MR) approach. Utilizing genetic data from five UKB datasets, one IEU dataset, one EBI dataset encompassing traits linked to anxiety and depressive states, and tinnitus data sourced from the FinnGen project, we conducted two-sample MR analyses. Instrumental variables were selected based on stringent criteria, including genome-wide significance, clumping to ensure independence, and the exclusion of palindromic Single Nucleotide Polymorphisms and those associated with confounders. The primary MR analysis employed the Inverse Variance Weighted method, supplemented by sensitivity analyses using the Weighted Median and MR-Egger methods, to address potential pleiotropy. MR analyses suggested a genetic correlation between anxiety-depression and an increased risk of tinnitus. These findings were robust across various sensitivity analyses, including MR-Egger and MR-PRESSO, which supported the absence of pleiotropy and outliers. No evidence of reverse causality was found, strengthening the argument regarding the unidirectional influence of psychological factors on tinnitus. Our results indicate that a genetic predisposition to anxiety and depression can significantly enhance the risk of developing tinnitus. This finding the integration of psychological assessments and interventions in the management of tinnitus, highlighting the importance of addressing mental health components in auditory conditions. Further studies are required to explore these associations in more diverse populations and refine the mechanisms underlying these relationships.

**Supplementary Information:**

The online version contains supplementary material available at 10.1007/s12070-025-05618-x.

## Introduction

Tinnitus is identified as a hallucinatory perception occurring in individuals without any external stimulus, thus representing a cognitive anomaly [1]. Reports indicate that the prevalence of tinnitus in the US was 11.2% (95% CI 10.8, 11.7%; ∼27 million people) in 2014. [2] For a minority of individuals (5–8%) [3], tinnitus causes significant distress and becomes debilitating. However, the correlation between tinnitus and stress and anxiety states cannot be clearly obtained from questionnaire studies alone, and it is not known whether anxiety and depression induce tinnitus. Consequently, identifying the key risk factors for tinnitus is essential for improving diagnosis, treatment, and patient quality of life.

Earlier clinical studies on the link between anxiety-depression and tinnitus have shown that patients are significantly more likely to exhibit psychopathological symptoms including somatization, phobias, anxiety, depression, and paranoid ideation (*P* < 0.05) [4–6]. However, the specific connection between anxiety-depression and tinnitus requires further investigation. This raises the question of whether there is a causal relationship between anxiety-depression and tinnitus? Is there a mediator between anxiety, depression, and tinnitus that connect them? This study aimed to investigate potential causal connections using Mendelian randomization, providing genetic insight into the association.

Mendelian randomization (MR) is a method used to explore causal relationships between exposure and desired outcomes. This technique employs single nucleotide polymorphisms (SNPs) as unbiased proxies for such exposures, effectively bypassing the typical residual confounding and reverse causality issues found in standard observational studies. Owing to the lack of randomized clinical trials (RCTs), MR is considered a crucial approach for inferring causality. This is because genetic variants are randomly distributed during meiosis, which closely simulates the random assignment process in an RCT. We performed a two-sample Mendelian randomization (MR) study to examine the impact of an anxiety-depression on the risk of tinnitus. We used genetic variants linked to this psychological condition as instrumental variables (IVs) to determine their causal influence on tinnitus. Moreover, we explored the relationship between anxiety-depression and tinnitus. These investigative approaches were chosen to deepen our understanding of the role of depression in patients with tinnitus. Considering the global variability observed, this could have important implications for both clinical practice and public health policies.

## Method

### Study Design

**Fig. 1:**
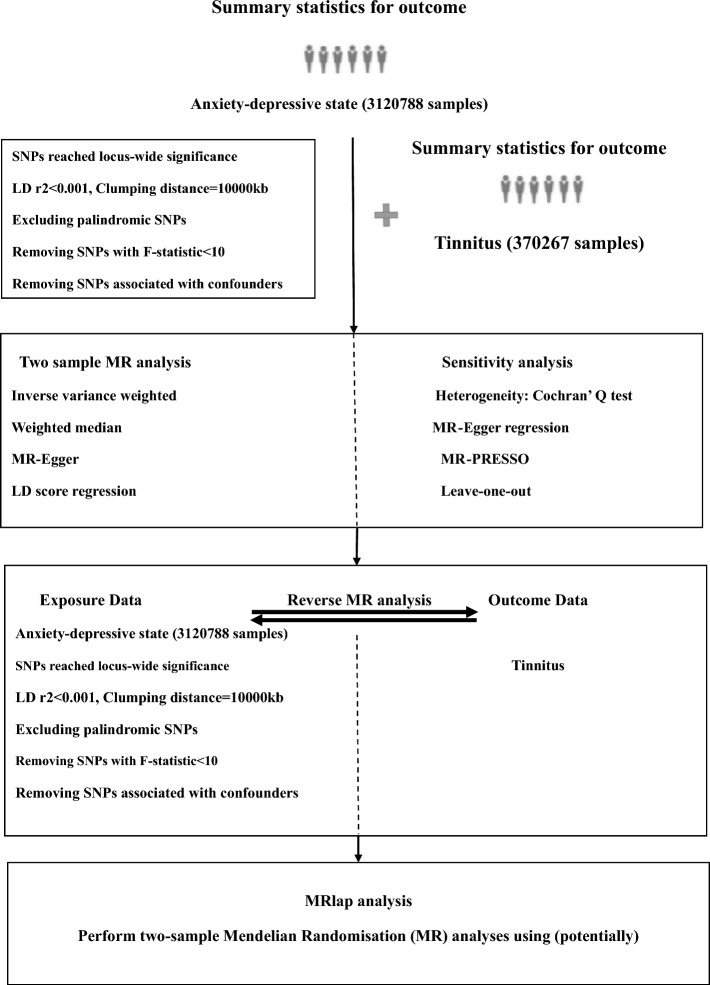
The study design and major assumptions of the 2-sample MR study. MR = Mendelian randomization

In this study, two-sample Mendelian Randomization (MR) was utilized to examine the relationship between anxiety-depression and tinnitus, with the study design illustrated in Fig. 1. For the MR results to be considered valid, three critical conditions need to be satisfied: (I) The instrumental variables (IVs) used in the analysis must have a strong association with exposure. (II) These IVs must not be linked to confounders that could influence both exposure and outcome. (Sleep disturbances, alcohol intake, etc.) Sleep and alcohol consumption are considered to be important independent factors of tinnitus [7]. (III) The impact of IVs on the outcome should be mediated exclusively through exposure without any alternative pathways [8].

### Linkage Disequilibrium Score Regression

The LDSC operates fundamentally as a linear regression model where the input consists of the IEU analysis results. The magnitude of heritability can also be assessed using LDSC [9].

Before conducting Mendelian randomization, LDSC was employed to examine the genetic link between anxiety, depression and tinnitus. This was complemented by a review of existing literature on the relationship between anxiety, depression, and tinnitus.

### Mendelian Randomization Analysis

In this study, the Inverse Variance Weighted (IVW) method was primarily utilized to determine the causal link between anxiety-depression and tinnitus, assuming the absence of horizontal pleiotropy [10]. Two distinct sets of genetic instruments were employed to elucidate the causality of anxiety-depression affecting tinnitus. To this end, we searched seven datasets related to anxiety and depressive states in the IEU namely: ukb-b-929, ukb-b-5664, ukb-b-6519, ukb-b-9981, ieu-b-102, ebi-a-GCST006948, and ukb-b-19809. The basic characteristics of the anxiety and depression databases are presented in Table S1. The principal components were corrected for each data point.

We randomized these seven datasets with the tinnitus dataset separately for two-sample Mendelian randomization (*P* < 5 × 10^–8^, linkage disquilibrium [LD] r^2^ < 0.001, Kb = 10,000). The F-statistics of each of these seven datasets were larger than the conventional value of 10, indicating that the instruments had a strong potential to predict tinnitus in subjects.

### IEU Summary Data for Tinnitus

Summary data for Tinnitus was sourced from the finngen_R10_H8_TINNITUS dataset, which included 7,914 cases of Tinnitus and 362,353 controls. We obtained summary data from the Practical database, extracting information on each SNP linked to tinnitus and those associated with the anxiety-depression, including their effects on tinnitus, effect sizes, and standard errors.

### Reverse-Direction Mendelian Randomization Analysis

Furthermore, we conducted reverse Mendelian Randomization using the IVW method on the anxiety-depression to assess whether tinnitus had a causal impact on anxiety-depression.

We randomly assigned this tinnitus dataset to each of the seven datasets, and owing to the insufficient number of SNPs extracted from the tinnitus dataset, the screening conditions for SNPs were adjusted for bivariate double-sample Mendelian randomization (*P* < 5 × 10^–6^, linkage disquilibrium [LD]r^2^ < 0.001, Kb = 10,000). When the *P*-value was 5e-08, the number of SNPs extracted from the exposure factor (tinnitus) for reverse Mendelian randomization was 0. When the *P*-value was 5e-07, the number of SNPs extracted from the exposure factor (tinnitus) of reverse Mendelian randomization was 3, and in further extraction, the number of SNPs was significantly insufficient for further analysis, so 5e-06 was selected as the extraction standard. The F-statistic in the tinnitus dataset was greater than the conventional value of 10, indicating that these instruments have a strong potential to predict anxiety-depression. The procedures for conducting reverse Mendelian Randomization mirrored those for standard Mendelian Randomization, as illustrated in Fig. 1.

### Statistical Analyses

We harmonized the effect alleles from IEU data on Anxiety-depression and Tinnitus before applying several MR methodologies to compute MR estimates for the influence of Anxiety-depression on Tinnitus. These include the IVW, weighted median, and MR-Egger methods. Each method addresses horizontal pleiotropy differently, which is why multiple techniques were employed.

Sensitivity analysis plays a vital role in identifying pleiotropy and addressing the heterogeneity that could substantially affect MR estimates. We used heterogeneity, indicated by a Cochran Q-derived p-value of less than 0.05 from the IVW method, to suggest potential horizontal pleiotropy. Directional pleiotropy was evaluated using the intercept from the MR-Egger regression, where a p-value below 0.05 confirmed its presence. Additionally, the MR-Pleiotropy Residual Sum and Outlier (MR-PRESSO) method was employed to assess and correct for horizontal pleiotropy. This approach involves detecting horizontal pleiotropy, rectifying it through outlier removal, and assessing significant shifts in causal estimates pre and post-correction. MR-PRESSO is notably less prone to bias and is more precise than both IVW and MR-Egger, particularly when fewer than 10% of the variants demonstrate horizontal pleiotropy. Leave-one-out analysis was also performed to check if any individual SNP disproportionately affected the MR estimates.

All statistical analyses in this study were conducted using the ‘TwoSampleMR’ package in the R software environment (specifically Version 4.3.3). For the MR-PRESSO tests, which can be thought of as the cleanup crew ensuring the data isn’t muddied by outliers, we utilized the dedicated ‘MR-PRESSO’ package. This setup ensured that our analyses were both robust and reliable, leveraging the specific strengths of each package to obtain the best possible insights from our data.

### MRlap Analysis

MRlap is an R-package tailored for performing two-sample Mendelian Randomization (MR) studies that may involve overlapping samples using only IEU summary data. The reliability of MR estimates can be compromised by several biases, such as overlapping samples between exposure and outcome, employment of weak instruments, and the winner’s curse phenomenon. We developed a method (MRlap) that simultaneously considers weak instrument bias and winner’s curse while accounting for potential sample overlap. Assuming spike‐and‐slab genomic architecture and leveraging linkage dis-equilibrium score regression and other techniques, we can analytically derive, reliably estimate, and hence correct for the bias of IVW‐MR using association summary statistics only. We tested our approach by using simulated data for a wide range of realistic settings. In all explored scenarios, our correction reduced the bias,in some situations by as much as 30‐fold [11].

### Data Sources

IEU summary data for Anxiety-depression was sourced from the ieu open IEU project, accessible via https://IEU.mrcieu.ac.uk/. Similarly, IEU data for tinnitus were acquired from FinnGen, which encompassed 7,914 cases and 362,353 controls from the R10_manifest cohort. These data are available at https://www.finngen.fi/en/access_results.

### Selection of Instruments Variables

To select appropriate instrumental variables (IVs), we performed comprehensive quality checks on single nucleotide polymorphisms (SNPs) as follows: (I) SNPs linked to exposure were chosen based on a genome-wide significance threshold (*P* < 5 × 10^-8^). (II) Clumping procedures (r^2^ < 0.001, clumping distance = 10,000 kb) were implemented to prevent linkage disequilibrium among IVs associated with anxiety-depression, ensuring the independence of SNPs. (III) We did not substitute SNPs missing in the outcome IEU with proxy SNPs that had a high linkage disequilibrium (R^2^ > 0.8) with the intended SNPs. (IV) Palindromic SNPs were excluded to maintain consistency in the effects of SNPs on both exposure and outcomes. (V) To remove SNPs potentially linked to outcome-related risk factors, we employed R language software to screen out SNPs associated with potential confounders such as sleep disturbances and alcohol intake and so on. [7] (VI) To combat the problem of weak IVs, we calculated the F-statistic for each SNP, where R^2 indicates the variance in exposure explained by the SNPs, n denotes the sample size, and k is the number of instrumental variables used. IVs with an F-statistic below 10 were considered weak and excluded from the analysis. F = R^2^ × (n-k-1)/k × (1-R^2^).

#### Ethics

The summary-level data used in this study were publicly available and de-identified with prior approval from the Ethical Standards Committee. No further ethical approval was obtained for this study.

## Results

### Selection of IVs Related to Anxiety-Depression

**Fig. 2:**
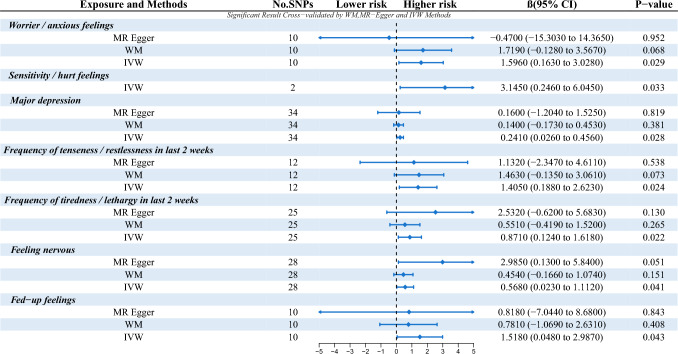
P-value, and beta results of causal links between anxiety and depressive state and tinnitus

In this study, we extensively searched for diseases related to emotional anxiety and depression in the IEU database, screened SNPS by significance threshold, excluded SNPS that might be related to confounders, and identified significant diseases related to anxiety and depression, including worrier/anxious feelings, sensitivity/hurt feelings, anxiety-depression, frequency of tiredness/lethargy in last 2 weeks,frequency of tenseness/restlessness in last 2 weeks, feeling nervous and fed-up feelings (Table 1 and Fig. 2).

**Table 1:** Significant MR analysis results of causal links between Anxiety-depressive state and Tinnitus

Anxiety-depressive state	Id	N SNPs	Traits	Method	OR	OR (95%CI)	Beta	*P*-value	MR- Egger Regression		Heterogeneity (IVW)		R2	F
									Egger Intercept	*P*-value	Cochran’s Q	*P*-value		
Worrier / anxious feelings	ukb-b-6519	10	Tinnitus	MR Egger	0.626	2.260e-07- 1.732e + 06	-0.469	0.952	0.016	0.791	7.292	0.607	0.0003	11.662
Worrier / anxious feelings	ukb-b-6519	10	Tinnitus	Weighted median	5.580	8.797e-01- 3.540e + 01	1.719	0.068						
Worrier / anxious feelings	ukb-b-6519	10	Tinnitus	Inverse variance weighted	4.931	1.177e + 00- 2.065e + 01	1.596	0.029						
Sensitivity / hurt feelings	ukb-b-9981	2	Tinnitus	MR Egger	–		–	–	NA	NA	0.135	0.713	5.9731E-05	13.423
Sensitivity / hurt feelings	ukb-b-9981	2	Tinnitus	Weighted median	–		–	–						
Sensitivity / hurt feelings	ukb-b-9981	2	Tinnitus	Inverse variance weighted	23.229	1.279–421.822	3.145	0.033						
Major depression	ieu-b-102	34	Tinnitus	MR Egger	1.174	0.300–4.595	0.160	0.819	0.002	0.907	34.254	0.407	0.0116	172.336
Major depression	ieu-b-102	34	Tinnitus	Weighted median	1.150	0.841–1.573	0.140	0.381						
Major depression	ieu-b-102	34	Tinnitus	Inverse variance weighted	1.273	1.026–1.578	0.241	0.028						
Frequency of tenseness / restlessness in last 2 weeks	ukb-b-5664	12	Tinnitus	MR Egger	3.102	0.096–100.631	1.132	0.538	0.003	0.873	7.446	0.762	0.0004	13.486
Frequency of tenseness / restlessness in last 2 weeks	ukb-b-5664	12	Tinnitus	Weighted median	4.320	0.874–21.359	1.463	0.073						
Frequency of tenseness / restlessness in last 2 weeks	ukb-b-5664	12	Tinnitus	Inverse variance weighted	4.077	1.207–13.774	1.405	0.024						
Frequency of tiredness / lethargy in last 2 weeks	ukb-b-929	25	Tinnitus	MR Egger	12.574	0.538–293.760	2.532	0.129	-0.020	0.299	35.579	0.060	0.0014	25.331
Frequency of tiredness / lethargy in last 2 weeks	ukb-b-929	25	Tinnitus	Weighted median	1.734	0.658–4.572	0.551	0.265						
Frequency of tiredness / lethargy in last 2 weeks	ukb-b-929	25	Tinnitus	Inverse variance weighted	2.390	1.132–5.045	0.871	0.022						
Feeling nervous	ebi-a-GCST006948	28	Tinnitus	MR Egger	19.786	1.139–343.731	2.985	0.051	-0.038	0.103	43.219	0.025	0.0028	37.057
Feeling nervous	ebi-a-GCST006948	28	Tinnitus	Weighted median	1.574	0.847- 2.927	0.454	0.151						
Feeling nervous	ebi-a-GCST006948	28	Tinnitus	Inverse variance weighted	1.764	1.024–3.040	0.568	0.041						
Fed-up feelings	ukb-b-19809	10	Tinnitus	MR Egger	2.266	0.001–5882.128	0.818	0.843	0.006	0.863	10.859	0.286	0.0003	12.477
Fed-up feelings	ukb-b-19809	10	Tinnitus	Weighted median	2.184	0.343–13.893	0.781	0.408						
Fed-up feelings	ukb-b-19809	10	Tinnitus	Inverse variance weighted	4.561	1.049–19.833	1.518	0.043						

N snps, number of single nucleotide polymorphism; OR, odds ratio

### Causal Effects and Sensitivity Analysis of Anxiety-Depression on Tinnitus

The initial MR analysis confirmed a link between anxiety-depression and the risk of tinnitus, with all instrumental variables (IVs) demonstrating strong instrument strength (*F* > 10), as detailed in Table 1. Subsequent analyses and sensitivity assessments predominantly identified significant outcomes associated with anxiety-depression. Notably, elevated levels of Worrier / anxious feelings, Sensitivity / hurt feelings, Major depression, Frequency of tiredness / lethargy in last 2 weeks, Frequency of tenseness / restlessness in last 2 weeks, Feeling nervous and Fed-up feelings correlated with increased tinnitus risk [odds ratio (OR): 4.913, 95% confidence interval (CI) 1.177e + 00–2.065e + 01, *P* = 0.029 for Worrier / anxious feelings; OR 23.229, 95% CI 1.279–421.822, *P* = 0.033 for Sensitivity / hurt feelings; OR 1.273, 95% CI 1.026–1.578, *P* = 0.028 for Major depression; OR 4.077, 95% CI 1.207–13.774, *P* = 0.024 for Frequency of tenseness / restlessness in last 2 weeks; OR 2.390, 95% CI 1.132–5.045, *P* = 0.022 for Frequency of tiredness / lethargy in last 2 weeks; OR: 1.764, 95% CI 1.024–3.040, *P* = 0.041 for Feeling nervous and OR 4.561, 95% CI 1.049–19.833, *P* = 0.043 for Fed-up feelings], as indicated in Table 1 and Fig. 2.

Next, we extracted SNPS from seven anxiety and depression related databases in the exposure group to form a dataset with 121 SNPs; after removing confounding factors (alcohol intake) after sleep disorders), 109 SNPs remained, which were searched for the potential outcome factors of each SNP in the GWAS database of the whole group, and 11,455 related phenotypes were obtained, and 2212 related phenotypes remained after removing duplicates. In addition, we screened the exposure factors at the GWAS level in the tinnitus dataset, and a total of 441 tinnitus-related exposure factors were obtained, and 441 tinnitus-related exposure factors were interfaced with 2212 outcome factors of anxiety and depression states. By checking each possibility in the PUBMED database, the cognitive performance and body weight indicators were inferred as mediating variables between anxiety and depression and tinnitus disease development (Supplementary Document 2).

### MRlap Analysis to Identify Anxiety-Depression on Tinnitus

MRlap estimates can be affected by several biases such as overlapping samples between exposure and outcome, the use of weak instruments, and the winner’s curse. Results from the MRlap test indicated no significant overlap between exposure and outcome factors, suggesting that errors from overlap rates were minimal and that IVW-MR estimates could be reliably utilized. (Table 2).

**Table 2:** MRlap analysis to identify Anxiety-depressive state on Tinnitus

Anxiety-depressive state	Id	Sample	MR correction observed effect	MR correction observed effect se	MR correction m IVs	MR correction observed effect p	MR correction corrected effect	MR correction corrected effect se	MR correction corrected effect p	MR correction test difference	MR correction p difference
Worrier / anxious feelings	ukb-b-6519	450,765	0.082	0.0218	84	0.0002	0.109	0.028	0.0001	− 4.304	1.6801e-05
Sensitivity / hurt feelings	ukb-b-9981	449,419	0.100	0.0306	50	0.0010	0.136	0.040	0.0008	− 3.505	0.0005
Major depression	ieu-b-102	500,199	0.111	0.0278	66	6.5236e-05	0.149	0.037	4.7119e-05	− 4.087	4.3776e-05
Frequency of tenseness / restlessness in last 2 weeks	ukb-b-5664	445,194	0.098	0.0343	27	0.0044	0.139	0.048	0.0037	− 2.931	0.0034
Frequency of tiredness / lethargy in last 2 weeks	ukb-b-929	449,019	0.094	0.0278	47	0.0007	0.128	0.038	0.0007	− 3.379	0.0007
Fed-up feelings	ukb-b-19809	453,071	0.052	0.0253	85	0.0419	0.066	0.032	0.0408	− 1.819	0.0689
Feeling nervous	ebi-a-GCST006948	373,121	0.090	0.0325	42	0.0056	0.124	0.044	0.0045	− 2.768	0.0056

### LDSC Analysis to Identify Genetic Association Between Anxiety-Depression and Tinnitus

The LDSC fundamentally operates as a linear regression model using the IEU analysis results as input. In this model, the independent variable was the LD score of the SNP locus, whereas the dependent variable was the bespoke statistic adhering to a chi-square distribution. The magnitude of heritability can also be assessed useing LDSC. We examined the genetic association between seven disease states and tinnitus by LDSC: feeling nervous, worrier/anxious feelings, sensitivity/hurt feelings, anxiety-depression, Frequency of tiredness/lethargy in last two weeks, frequency of tenseness/restlessness in last two weeks and fed-up feelings (Table 3).

**Table 3:** LDSC analysis to identify Genetic association between Anxiety-depressive state and Tinnitus

Exposure	Id	outcome	h2 observed	h2 observed se	h2 Z	h2 p	rg	rg se	rg p
Feeling nervous	ebi-a-GCST006948	tinnitus	0.056	0.003	19.364	1.57E-83	0.215	0.061	0.0004
Worrier / anxious feelings	ukb-b-6519	tinnitus	0.069	0.004	17.248	1.16E-66	0.267	0.102	0.009
Sensitivity / hurt feelings	ukb-b-9981	tinnitus	0.054	0.003	20.343	5.35E-92	0.290	0.089	0.001
Major depression	ieu-b-102	tinnitus	0.056	0.002	22.748	1.50E-114	0.341	0.059	6.43E-09
Frequency of tenseness / restlessness in last 2 weeks	ukb-b-5664	tinnitus	0.039	0.002	16.077	3.72E-58	0.327	0.079	3.23E-05
Frequency of tiredness / lethargy in last 2 weeks	ukb-b-929	tinnitus	0.055	0.003	20.840	1.86E-96	0.192	0.065	0.003
Fed-up feelings	ukb-b-19809	tinnitus	0.064	0.003	21.849	7.93E-106	0.208	0.079	0.009

### Reverse Mendelian Randomization Analysis of Tinnitus and Anxiety and Depression

**Fig. 3:**
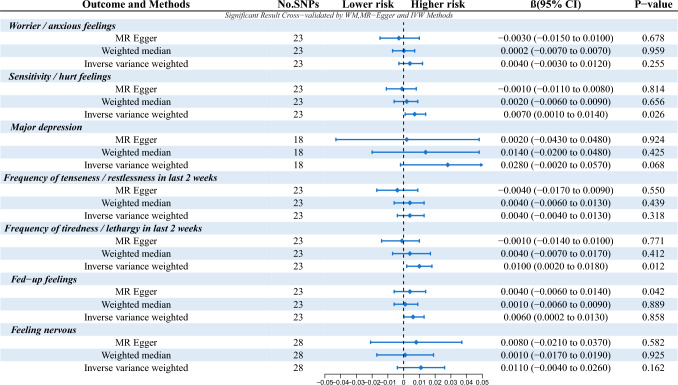
The P-value, and beta results results of reverse Mendelian randomization between anxiety and depressive state and tinnitus

The initial MR analysis confirmed a link between anxiety-depression and the risk of tinnitus, with all instrumental variables (IVs) demonstrating strong instrument strength (*F* > 10), as detailed in Table 4. Subsequent analyses and sensitivity assessments identified significant outcomes associated with tinnitus. The results are presented in Table 4 and Fig. 3.

**Table 4:** Reverse Mendelian randomization analysis of tinnitus and anxiety and depression

Traits	N SNPs	Anxiety-depressive state	Id	Method	OR	OR (95% CI)	Beta	*P*-value	MR- Egger Regression		Heterogeneity (IVW)		R^2^	F
									Egger Intercept	*P*-value	Cochran’s Q	*P*-value		
Tinnitus	23	Worrier / anxious feelings	ukb-b-6519	MR Egger	0.997	0.985–1.010	-0.003	0.678	0.001	0.167	67.072	1.895094e-06	0.073	1268.132
Tinnitus	23	Worrier / anxious feelings	ukb-b-6519	Weighted median	1.000	0.993–1.007	0.0002	0.959						
Tinnitus	23	Worrier / anxious feelings	ukb-b-6519	Inverse variance weighted	1.004	0.997–1.012	0.004	0.255						
Tinnitus	23	Sensitivity / hurt feelings	ukb-b-9981	MR Egger	0.999	0.989–1.008	-0.001	0.814	0.002	0.043	46.036	0.002		
Tinnitus	23	Sensitivity / hurt feelings	ukb-b-9981	Weighted median	1.002	0.994–1.009	0.002	0.656						
Tinnitus	23	Sensitivity / hurt feelings	ukb-b-9981	Inverse variance weighted	1.007	1.001–1.014	0.007	0.026						
Tinnitus	18	Major depression	ieu-b-102	MR Egger	1.002	0.958-1.049	0.002	0.924	0.005	0.176	36.314	0.004		
Tinnitus	18	Major depression	ieu-b-102	Weighted median	1.014	0.980–1.049	0.014	0.425						
Tinnitus	18	Major depression	ieu-b-102	Inverse variance weighted	1.028	0.998–1.059	0.028	0.068						
Tinnitus	23	Frequency of tenseness / restlessness in last 2 weeks	ukb-b-5664	MR Egger	0.996	0.983–1.009	-0.004	0.550	0.001	0.133	49.308	0.001		
Tinnitus	23	Frequency of tenseness / restlessness in last 2 weeks	ukb-b-5664	Weighted median	1.003	0.994–1.013	0.004	0.439						
Tinnitus	23	Frequency of tenseness / restlessness in last 2 weeks	ukb-b-5664	Inverse variance weighted	1.004	0.996–1.013	0.004	0.318						
Tinnitus	23	Frequency of tiredness / lethargy in last 2 weeks	ukb-b-929	MR Egger	0.998	0.986–1.010	-0.001	0.771	0.002	0.028	23.345	0.382		
Tinnitus	23	Frequency of tiredness / lethargy in last 2 weeks	ukb-b-929	Weighted median	1.005	0.993–1.017	0.004	0.412						
Tinnitus	23	Frequency of tiredness / lethargy in last 2 weeks	ukb-b-929	Inverse variance weighted	1.010	1.002–1.018	0.010	0.012						
Tinnitus	23	Fed-up feelings	ukb-b-19809	MR Egger	1.004	0.994–1.014	0.004	0.042	0.000					
	0.546	44.272	0.003											
Tinnitus	23	Fed-up feelings	ukb-b-19809	Weighted median	1.001	0.994–1.009	0.001	0.889						
Tinnitus	23	Fed-up feelings	ukb-b-19809	Inverse variance weighted	1.006	1.000–1.013	0.006	0.858						
Tinnitus	20	Feeling nervous	ebi-a-GCST006948	MR Egger	1.008	0.980-1.038	0.008	0.582	0.000	0.823	34.263	0.017		
Tinnitus	20	Feeling nervous	ebi-a-GCST006948	Weighted median	1.001	0.983-1.019	0.001	0.925						
Tinnitus	20	Feeling nervous	ebi-a-GCST006948	Inverse variance weighted	1.011	0.996-1.027	0.011	0.162						

N snps, number of single nucleotide polymorphism; OR, odds ratio

Studies have proved that tinnitus can promote anxiety and depression.

## Discussion

Tinnitus is a self-hearing sensation mediated by a variety of causes and is one of the most common ear conditions worldwide, with approximately 20% of patients suffering from or have suffered from tinnitus. The etiology of tinnitus is complex. Some studies have suggested that anxiety-depression are widespread in patients with tinnitus; however, whether there is a causal relationship between anxiety-depression and tinnitus has not been determined [12, 13].

Participants diagnosed with tube tinnitus, who had no prior psychiatric treatment history, exhibited significant depressive symptoms. As the severity of tinnitus increased, so did anxiety and neuroticism scores.

The occurrence of tinnitus is an extremely complex process that is affected by several factors. In recent years, with the proposed new biopsychosocial model, most doctors believe that tinnitus should be classified as a physical and mental disease, and that psychological factors play a decisive role in its occurrence and development. At the same time, most tinnitus patients also clearly pointed out that in addition to auditory symptoms, there are certain mental symptoms, such as anxiety and depression, during the onset of tinnitus [14, 15]. After the appearance of tinnitus symptoms, tinnitus sounds through the amygdala lead to fear in patients, and promote the hippocampus to produce fear emotion memory in tinnitus. This emotion generated through the amygdala subcortical pathway may be the key reason why patients with tinnitus experience anxiety and depression. The similar studies have confirmed that patients with tinnitus have unique mental traits that are closely related to anxiety and depression, and that this mental trait of anxiety and depression subtly affects tinnitus patients, thereby inducing tinnitus in patients [16–18], and that a lifetime history of mental illness and migraine is a predictor of disabling tinnitus.

Our study confirmed a causal relationship between anxiety-depression and tinnitus through Mendelian randomization of disorders associated with anxiety-depression and tinnitus. Seven diseases and states related to anxiety and depression can aggravate the occurrence of tinnitus and promote further progression of tinnitus.

Besides, we extracted SNPS from seven anxiety and depression related databases in the exposure group to form a dataset with 121 SNPS, after removing confounding factors (alcohol intake) After sleep disorders), 109 SNPS were remaining, which were searched for the potential outcome factors of each SNP in the GWAS database of the whole group, and 11,455 related phenotypes were obtained, and 2212 related phenotypes were remaining after removing duplicates In addition, we screened the exposure factors at the GWAS level in the tinnitus dataset, and a total of 441 tinnitus-related exposure factors were obtained, and 441 tinnitus-related exposure factors were interfaced with 2212 outcome factors of anxiety and depression states. By examining each possibility in the PUBMED database, the cognitive performance were finally inferred by mediating variables between anxiety and depression and tinnitus disease development, which included 14 snps, including rs2367724,rs2568958,rs34811474,rs4625,rs55997507,rs56327309,rs9879090,rs6919397,rs7241572,rs754287,rs8089865,rs9807529,rs17622606 and rs11971935. Tinnitus was associated with a decreased cognitive performance, especially on tasks measuring delayed memory [19].

This made it possible to treat tinnitus psychologically, and cognitive behavioral therapy came into being.

Our research presents seven extensive, large-scale Mendelian Randomization (MR) studies examining the genetic-level causal relationship between anxiety-depression and tinnitus. It leverages the most recent, comprehensive IEU data and employs genetic prediction techniques to establish this causality. In addition to utilizing the MR method, we also incorporated the MRlap approach, which accounts for and corrects various biases using cross-trait LD score regression (LDSC) to estimate sample overlaps. This corrected effect estimation was utilized as a sensitivity analysis: if there was no significant difference from the observed effect, the IVW-MR estimate was considered reliable.

Mendelian randomization is second only to RCTs in evidence-based medicine, and this study provides evidence-based support for the clinical exploration of the relationship between anxiety, depression and tinnitus.

However, our study has some limitations: First, the IEU data are exclusively from individuals of European descent, limiting the generalizability of our findings to other populations. Second, the use of the R language software to eliminate confounding factors (Sleep disorders, alcohol intake, etc.) may introduce bias due to subjective decisions by the authors, necessitating caution in interpreting the results. (III)We developed a method (MRlap) that simultaneously considers weak instrument bias and the winner’s curse, while accounting for potential sample overlap. Assuming a spike‐and‐slab genomic architecture and leveraging linkage dis-equilibrium score regression and other techniques, we can analytically derive, reliably estimate, and hence correct for the bias of IVW‐MR using association summary statistics only. We tested our approach by using simulated data for a wide range of realistic settings. In all the explored scenarios, our correction reduced the bias in some situations by as much as 30‐fold. While sample overlap might inflate test outcomes, we anticipate a minimal impact, as no explicit sample overlap was identified.

## Conclusion

This study established a causal link between an anxiety-depression and tinnitus, indicating that anxiety and depression may exacerbate tinnitus symptoms. However, no evidence of inverse causality was found. Additionally, these findings strengthen the evidence-based medical foundation concerning the relationship between tinnitus,anxiety, and depression. They offer theoretical support for future cognitive behavioral therapies aimed at treating tinnitus and suggest broader cognitive strategies for managing this condition.

## Supplementary Information

Below is the link to the electronic supplementary material.

Supplementary file1 (RAR 504 KB)

Supplementary file2 (RAR 1215 KB)

Supplementary file3 (RAR 4780 KB)

Supplementary file4 (DOCX 16 KB)

## Data Availability

The datasets presented in this study can be found in the online repositories. The names of the repository/repositories and accession numbers can be found in the article/supplementary material.

## References

[1] Dobel C, Junghöfer M (2024) Tinnitus-on the interplay between emotion and cognition. HNO 72(Suppl 1):46–50. 10.1007/s00106-023-01339-1. (**PMID: 37725160**) 37725160 10.1007/s00106-023-01339-1

[2] Batts S, Stankovic KM (2024) Tinnitus prevalence, associated characteristics, and related healthcare use in the United States: a population-level analysis. Lancet Reg Health Am 29:100659. 10.1016/j.lana.2023.100659. (**PMID:38269207; PMCID:PMC10806285**) 38269207 10.1016/j.lana.2023.100659 PMC10806285

[3] Cima RFF, Mazurek B, Haider H et al (2019) A multidisciplinary European guideline for tinnitus: diagnostics, assessment, and treatment. HNO 67(Suppl 1):10–42. 10.1007/s00106-019-0633-7. (**PMID: 30847513**) 30847513 10.1007/s00106-019-0633-7

[4] Fuller TE, Haider HF, Kikidis D et al (2017) A systematic review of existing clinical guidelines for the assessment and treatment of tinnitus in adults. Front Psychol 8:206. 10.3389/fpsyg.2017.00206. (**PMID:28275357;PMCID:PMC5319986**) 28275357 10.3389/fpsyg.2017.00206 PMC5319986

[5] Ahmed A, Aslam N (2022) Psychopathological symptoms as a common risk factor for tinnitus distress and magnitude: a cross-sectional study. J Pak Med Assoc 72(10):2034–2037. 10.47391/JPMA.4644. (**PMID: 36660973**) 36660973 10.47391/JPMA.4644

[6] Bhatt IS, Wilson N, Dias R, Torkamani A (2022) A genome-wide association study of tinnitus reveals shared genetic links to neuropsychiatric disorders. Sci Rep 12(1):22511. 10.1038/s41598-022-26413-6. (**PMID:36581688;PMCID:PMC9800371**) 36581688 10.1038/s41598-022-26413-6 PMC9800371

[7] Basso L, Boecking B, Brueggemann P et al (2020) Gender-specific risk factors and comorbidities of bothersome tinnitus. Front Neurosci 14:706. 10.3389/fnins.2020.00706.PMID:33071718;PMCID:PMC7539146 33071718 10.3389/fnins.2020.00706 PMC7539146

[8] Davey Smith G, Hemani G (2014) Mendelian randomization: genetic anchors for causal inference in epidemiological studies. Hum Mol Genet 23(R1):R89-98. 10.1093/hmg/ddu328. (**PMID: 25064373; PMCID: PMC4170722**) 25064373 10.1093/hmg/ddu328 PMC4170722

[9] Bulik-Sullivan BK, Loh PR, Finucane HK et al (2015) LD Score regression distinguishes confounding from polygenicity in genome-wide association studies. Nat Genet 47(3):291–295. 10.1038/ng.3211. (**PMID: 25642630; PMCID: PMC4495769**) 25642630 10.1038/ng.3211 PMC4495769

[10] Burgess S, Butterworth A, Thompson SG (2013) Mendelian randomization analysis with multiple genetic variants using summarized data. Genet Epidemiol 37(7):658–665. 10.1002/gepi.21758. (**PMID: 24114802; PMCID: PMC4377079**) 24114802 10.1002/gepi.21758 PMC4377079

[11] Mounier N, Kutalik Z (2023) Bias correction for inverse variance weighting Mendelian randomization. Genet Epidemiol 47(4):314–331. 10.1002/gepi.22522. (**PMID: 37036286**) 37036286 10.1002/gepi.22522

[12] Karaaslan Ö, Kantekin Y, Hacımusalar Y, Dağıstan H (2020) Anxiety sensitivities, anxiety and depression levels, and personality traits of patients with chronic tinnitus: a case-control study. Int J Psychiatry Clin Pract 24(3):264–269. 10.1080/13651501.2020.1757117. (**PMID: 32374199**) 32374199 10.1080/13651501.2020.1757117

[13] Caldirola D, Teggi R, Daccò S, Sangiorgio E, Bussi M, Perna G (2016) Role of worry in patients with chronic tinnitus and sensorineural hearing loss: a preliminary study. Eur Arch Otorhinolaryngol 273(12):4145–4151. 10.1007/s00405-016-4100-8. (**PMID: 27197727**) 27197727 10.1007/s00405-016-4100-8

[14] Pajor AM, Ormezowska EA, Jozefowicz-Korczynska M (2013) The impact of co-morbid factors on the psychological outcome of tinnitus patients. Eur Arch Otorhinolaryngol 270(3):881–888. 10.1007/s00405-012-2079-3. (**Epub 2012 Jun 15 PMID: 22699628**) 22699628 10.1007/s00405-012-2079-3

[15] Fagelson MA, Smith SL (2016) Tinnitus self-efficacy and other tinnitus self-report variables in patients with and without post-traumatic stress disorder. Ear Hear 37(5):541–546. 10.1097/AUD.0000000000000290. (**PMID: 26950001**) 26950001 10.1097/AUD.0000000000000290

[16] van Munster JJCM, van der Valk WH, Stegeman I, Lieftink AF, Smit AL (2020) The relationship of tinnitus distress with personality traits: a systematic review. Front Neurol 11:225. 10.3389/fneur.2020.00225. (**PMID:32655464;PMCID:PMC7326028**) 32655464 10.3389/fneur.2020.00225 PMC7326028

[17] Mucci S, Geocze L, Abranches DC, Antúnez AE, Penido Nde O (2014) Revisão sistemática sobre as evidências da associação entre personalidade e zumbido Systematic review of evidence on the association between personality and tinnitus. Braz J Otorhinolaryngol 80(5):441–447. 10.1016/j.bjorl.2014.05.031. (**PMID: 25303821 PMCID: PMC9444609**) 25303821 10.1016/j.bjorl.2014.05.031 PMC9444609

[18] Durai M, Searchfield G (2016) Anxiety and depression, personality traits relevant to tinnitus: a scoping review. Int J Audiol 55(11):605–615. 10.1080/14992027.2016.1198966. (**PMID: 27387463**) 27387463 10.1080/14992027.2016.1198966

[19] Cardon E, Vermeersch H, Joossen I et al (2022) Cortical auditory evoked potentials, brain signal variability and cognition as biomarkers to detect the presence of chronic tinnitus. Hear Res 420:108489. 10.1016/j.heares.2022.108489. (**PMID: 35354098**) 35354098 10.1016/j.heares.2022.108489

