# Supplementary material for: Causal Effects Between Anxiety-Depressive and Subjective Tinnitus in Europe: A Bidirectional Mendelian Randomization Study
Source: Indian J Otolaryngol Head Neck Surg. 2025 Jun 9;77(8):2979–91. doi: 10.1007/s12070-025-05618-x (PMC12297070; doi:10.1007/s12070-025-05618-x)
Supplement: Supplementary file 4 — Supplementary file4 (DOCX 16 KB) [file 12070_2025_5618_MOESM4_ESM.docx]

Table S1. Basic Characteristics of Anxiety and Depression states Databases in the study

| Data | Author | Dataset | Samplesize |
| --- | --- | --- | --- |
| Worrier / anxious feelings | Ben Elsworth et al | Ukb-b-6519 | 450,765 |
| Frequency of tiredness / lethargy in last 2 weeks | Ben Elsworth et al | Ukb-b-929 | 449,019 |
| Sensitivity / hurt feelings | Ben Elsworth et al | Ukb-b-9981 | 449,419 |
| Frequency of tenseness / restlessness in last 2 weeks | Ben Elsworth et al | Ukb-b-5664 | 445,194 |
| Fed-up feelings | Ben Elsworth et al | Ukb-b-19809 | 453,071 |
| Major depression | Howard DM et al | Ieu-b-102 | 500,199 |
| Feeling nervous | Nagel M et al | ebi-a-GCST006948 | 373,121 |
